# COVID-19 Vaccine mRNABNT162b2 Elicits Human Antibody Response in Milk of Breastfeeding Women

**DOI:** 10.3390/vaccines9070785

**Published:** 2021-07-13

**Authors:** Maurizio Guida, Daniela Terracciano, Michele Cennamo, Federica Aiello, Evelina La Civita, Gennaro Esposito, Valentina Gargiulo, Giuseppe M. Maruotti, Giuseppe Portella, Laura Sarno

**Affiliations:** 1 Department of Neurosciences, Reproductive Science and Dentistry, University of Naples “Federico II”, 80131 Naples, Italy; mauguida@unina.it (M.G.); federichella91@gmail.com (F.A.); gm.mar@tiscali.it (G.M.M.); laurettasarno@gmail.com (L.S.); 2 Department of Translational Medical Sciences, University of Naples “Federico II”, 80131 Naples, Italy; michelecennamo1@gmail.com (M.C.); eva.lacivita@gmail.com (E.L.C.); portella@unina.it (G.P.); 3 Department of Public Health, University of Naples “Federico II”, 80131 Naples, Italy; gennaroesposito17@gmail.com; 4 Department of Mother and Child, University Hospital Federico II, 80131 Naples, Italy; valegargiulo@yahoo.it

**Keywords:** vaccine, milk, immunoglobulins, SARS-Cov-2

## Abstract

Objective: The objective of this research is to demonstrate the release of SARS-CoV-2 Spike (S) antibodies in human milk samples obtained by patients who have been vaccinated with mRNABNT162b2 vaccine. Methods: Milk and serum samples were collected in 10 volunteers 20 days after the first dose and 7 seven days after the second dose of the mRNABNT162b2 vaccine. Anti-SARS-CoV-2 S antibodies were measured by the Elecsys^®^ Anti-SARS-CoV-2 S ECLIA assay (Roche Diagnostics AG, Rotkreuz, Switzerland), a quantitative electrochemiluminescence immunometric method. Results: At first sample, anti-SARS-CoV-2 S antibodies were detected in all serum samples (103.9 ± 54.9 U/mL) and only in two (40%) milk samples with a low concentration (1.2 ± 0.3 U/mL). At the second sample, collected 7 days after the second dose, anti-SARS-CoV-2 S antibodies were detected in all serum samples (3875.7 ± 3504.6 UI/mL) and in all milk samples (41.5 ± 47.5 UI/mL). No correlation was found between the level of serum and milk antibodies; the milk antibodies/serum antibodies ratio was on average 2% (range: 0.2–8.4%). Conclusion: We demonstrated a release of anti-SARS-CoV-2 S antibodies in the breast milk of women vaccinated with mRNABNT162b2. Vaccinating breastfeeding women could be a strategy to protect their infants from COVID-19 infection.

## 1. Introduction

One year after the beginning of the COVID-19 pandemic, the COVID-19 vaccine was introduced in European countries as an important gamechanger in the battle against COVID-19. The European Medicine Agency (EMA) has currently authorized four different vaccines: Pfizer/BioNtech Comirnaty mRNABNT162b2 and Moderna mRNA-1273, that are COVID-19 mRNA vaccines; COVID-19 Vaccine AstraZeneca (*ChAdOx1-S [recombinant]*) and Janssen (Johnson & Johnson) (*Ad26.COV2-S (recombinant)*), that are recombinant vaccines (https://www.ema.europa.eu/en/human-regulatory/overview/public-health-threats/coronavirus-disease-COVID-19/treatments-vaccines/vaccines-COVID-19/COVID-19-vaccines-authorised#authorised-COVID-19-vaccines-section accessed on 27 March 2021).

Before the beginning of the vaccination campaign in Europe, clinical trials for the COVID-19 vaccines currently authorised for use did not include people younger than 16 years old (for mRNABNT162b2) or 18 years old (for the others), pregnant women, and people who were breastfeeding [1]. Very recently, data on the safety of the vaccine also in adolescents (older than 12 years old) [2] and pregnant women [3] have been published.

Considering that the COVID-19 vaccines currently used are not based on replication-competent viruses and other non-replicating viral vaccines are safe during breastfeeding, the Centers for Disease Control and Prevention (CDC) stated that lactating women may choose to be vaccinated [4].

Similarly, the Academy of Breastfeeding Medicine (ABM) [5], the American College of Obstetrics and Gynecology (ACOG) [6], and the Royal College of Obstetrics and Gynecology (RCOG) [7] recommended that COVID-19 vaccines should be offered to lactating individuals.

It has been reported that COVID-19 antibodies can be detected in patients that were infected by COVID-19, and they could provide passive immunity to breastfed infants [6].

Data related to the presence of SARS-CoV-2 antibodies in breastmilk are still very limited.

The aim of our study was to demonstrate the release of SARS-CoV-2 Spike (S) antibodies in human milk samples obtained by patients who have been vaccinated with the mRNABNT162b2 vaccine.

## 2. Materials and Methods

This was a cohort prospective observational study. We included volunteers vaccinated with the mRNABNT162b2 vaccine despite breastfeeding. A previous SARS-CoV-2 infection was considered exclusion criteria. Only patients with a known negative serology for SARS-CoV-2, performed not more than 1 week before the first injection of vaccine, were included. We also collected samples by a negative control (with negative serology and not being vaccinated). All patients signed a written consent form, and the study has been approved by the local Ethical Committee (140/20/ES2COVID19). All information regarding human material was managed using anonymous numerical codes, and all samples were handled in compliance with the Helsinki Declaration (http://www.wma.net/en/30publications/10policies/b3/ accessed on 27 March 2021).

A questionnaire was administered to collect patients characteristics and length of breastfeeding. All data were recorded on a dedicated database. Data entering was double-checked by L.S and D.T.

A blood sample and a milk sample were collected twice: the day before (T1) and 7 days after the second dose of vaccine (T2).

Human milk samples of 4 mL were collected with clean breast pumps into sterile plastic containers and stored at −80 °C.

Patients were asked to collect milk samples before breastfeeding. Therefore, foremilk was collected, which is watery and with a lower fat concentration. All samples were collected in the morning, just before the appointment we had planned for the blood sample.

Blood samples were collected using a BD vacutainer (Becton Dickinson, Oxfordshire, UK) blood collection red tube (with no additives). After centrifugation, the serum was immediately stored at −80 °C.

Milk samples were thawed at 37 °C in a water bath for 10 min and centrifuged at 1300× *g* at 4 °C for 20 min to obtain delipidation. The solidified lipid layer was pierced with a pipette, and the soluble phase was carefully withdrawn. If necessary, centrifugation was repeated, and the clear supernatants were used for the detection of anti-SARS-CoV-2 S antibodies.

Milk and serum samples were tested using the Elecsys^®^ Anti-SARS-CoV-2 S ECLIA assay (Roche Diagnostics AG, Rotkreuz, Switzerland) performed on the Cobas e 801 analyzer (Roche Diagnostics) according to the manufacturers’ instruction, as already reported in a previous study [8]. This assay (Ref. # 09289275190) is a quantitative electrochemiluminescence immunometric method that assesses the concentration of a subject’s total immunoglobulin using a recombinant S protein (receptor binding domain) of SARS-CoV-2. Anti-SARS-CoV-2 S (total antibodies) were measured automatically by the analyser, which compares the electrochemiluminescence signal obtained from the reaction product of the sample with the signal previously obtained by calibration. The dynamic range of the assay is 0.40–250 U/mL. For values higher than 250 U/mL, the analyser automatically makes a 1:10 dilution.

Population characteristics were summarised by a descriptive statistic. Data were presented as mean ± standard deviation for continuous variables and number (percentage) for categorical variables. Correlation between antibody title in serum and milk was assessed by Spearman coefficient.

Statistical analysis was performed using SPSS 26.0 (SPSS Inc., Chicago, IL, USA).

## 3. Results

Ten women gave their consent to participate in the study. Characteristics of the study population are summarised in Table 1. All included women were Italian White. The mean age was 34.8 ± 4.2 years. The mean duration of breastfeeding at the time of the first dose of vaccine was 11 ± 5.1 months. The last pregnancy was singleton in all cases. All included women followed the recommended schedule for the mRNABNT162b2 vaccine (first dose at day 0 and second dose after 21 days). For 5 out of 10 women, we were able to collect serum and milk samples before and after the second dose of vaccine, while, for the remaining five, we collected both samples only 7 days after the second dose.

At T1, anti-SARS-CoV-2 S antibodies were detected in all serum samples (103.9 ± 54.9 U/mL) and only in two (40%) milk samples with a low concentration (1.2 ± 0.3 U/mL).

Serum antibody levels were higher in women with detectable anti S antibodies in milk samples; however, the difference was not significant (149 ± 26.9 U/mL vs. 73.8 ± 47.8 U/mL; *p =* 0.145).

**Figure 1:**
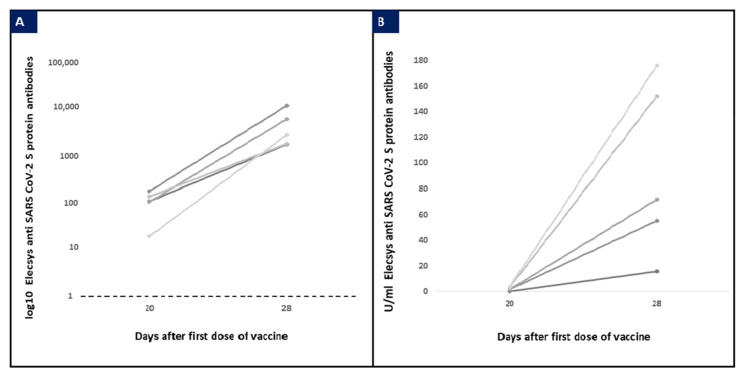
Log10 anti-SARS-CoV 2 S protein antibodies in maternal serum (**A**) and U/mL anti-SARS-CoV 2 S protein antibodies values in milk samples (**B**) in patients tested 20 and 28 days after the first dose (*n* = 5).

At T2, anti-SARS-CoV-2 S antibodies were detected in all sera (3875.7 ± 3504.6 UI/mL) and in all milk samples (41.5 ± 47.5 UI/mL). Anti-SARS-CoV-2 antibodies values in serum and milk samples are reported in Figure 1. No correlation was found between the level of serum and milk antibodies; milk antibodies/serum antibodies ratio was on average 2% (range: 0.2–8.4%). All patients received the second dose 21 days after the first one. The horizontal dotted line represents the cut-off of Elecsys immunoassay.

## 4. Discussion

Before the beginning of the vaccination campaign in Europe, the currently authorised vaccines have not been tested in specific subgroups such as pregnant and lactating women and, therefore, these categories were initially excluded from the vaccination campaign. While it has been established that SARS-CoV-2 RNA is not present in the breast milk of infected patients [9] and that in case of natural infection SARS-CoV-2 S antibodies are released in human milk [10], nowadays, only a few data are available about the effects of COVID-19 vaccines on breastfed infants. In our study, we analysed the presence of SARS-CoV-2 S antibodies in human milk samples of a cohort of 10 volunteers who were fully vaccinated with two doses of the COVID-19 vaccine mRNABNT162b2. We demonstrated the presence of antibodies in all samples of milk collected 7 days after the second dose. However, milk samples collected after the first dose did not show detectable SARS-CoV-2 antibodies in more than half of the cases, and, when present, the levels were very low. This timing is in agreement with the immunogenicity of mRNABNT162b2 vaccine, which showed a dose-dependent S protein-specific IgG increase [11].

Considering that we observed a significant increase in the serum level of antibodies after the second dose (up to 137 times), it is possible to speculate that it is necessary to reach threshold levels for the release of antibodies in human milk. However, we did not find a correlation between serum and milk antibodies. This can be related to the fact that many factors can affect antibodies’ concentration in human milk. Ig G and Ig A concentrations change across lactation, and it is higher in late breastfeeding [12]. Women included in our study have a different length of lactation, ranging from 3 to 20 months, and this variability could affect the results.

In this study, we used a SARS-CoV-2 anti-S assay ideally designed to detect IgA, IgM, and IgG, that predominantly reveals IgG [13], suggesting that the post-vaccine milk probably contains a majority of IgG. In previous studies, an IgA-dominant response has been observed in the breast milk of previously infected women [14,15].

This observation might be based on the different exposure routes of the mothers to viral antigen: the vaccinated via intramuscular injection, the infected via the mucosa, thus in the second group, the IgA play a pivotal role.

We can speculate that vaccine-specific response was not overlooked by IgA, but there is a time-dependent increase of IgG/IgA ratio, according to what has also been observed after Respiratory Syncytial Virus (RSV) infection [16].

Moreover, data from the study by Perl et al., demonstrating a spike in IgG 1 week after the second dose, is consistent with our results [8]. Gray et al. also demonstrated an increase of IgG levels after the second dose, while such a boost was not observed for IgM and IgA [17].

Unfortunately, another study [18] did not report anti-SARS-CoV2 levels before the second dose.

The main limitation of this study is the small sample size that makes it difficult to evaluate any statistical correlation or any trends in breastmilk antibody concentrations in association with maternal characteristics. Moreover, we included only women vaccinated with the BNT162b2 vaccine. However, since data on this topic is very limited, and some previous studies also reported on a small group of lactating women [18], we think our results can support the already known evidence. The range of durations of lactation for included patients is wide, making data difficult to generalise. Other limitations are related to the methodology: we did not characterise immunoglobulins subtypes in breast milk, and we did not evaluate the fraction of neutralising antibodies in breastmilk by in vitro competitive-blocking assay. Finally, the short duration of the follow-up period after the second dose of vaccine does not allow the assessment of the durability of the milk antibodies responses.

However, if confirmed, the predominance of IgG in breastmilk is particularly relevant since it has been already demonstrated their key role in neonatal immunity against other pathogens target of vaccines such as influenza and RSV [19].

To the best of our knowledge, this is the first European study demonstrating the release of SARS-CoV-2 S antibodies in human milk of breastfeeding women after COVID-19 vaccine mRNABNT162b2. Even if data are very limited and we cannot conclude that antibodies found can have a role in protecting newborns against COVID-19 infection based on our results, demonstrating the presence of neutralising antibodies in breast milk can support future studies addressing the type, the neutralising ability, and the duration of antibody response in human milk, which will allow better clinical management of this very relevant issue.

## Figures and Tables

**Table 1:** Main characteristics and antibodies levels in serum and milk samples of the study population.

	Age	Gravidity	Parity	Gestational Age at Delivery (Weeks)	Complication during Pregnancy	Chronic Conditions	Lenght of Breastfeeding (Months)	Anti SARS-CoV2 T1 UI/mL (Serum)	Anti-SARS CoV2 T2 UI/mL (Serum)	Anti SARS CoV2 T1 UI/mL (Milk)	Anti SARS CoV2 T2 UI/mL (Milk)
**Patient 1**	34	2	0	33	Severe preeclampsia Abruptio placenta	None	5	103.8	1615	<0.4	15.5
**Patient 2**	30	1	0	32	pPROM and preterm labour	None	12	168	10,879	0.97	39.9
**Patient 3**	32	1	0	39	None	None	16	99	5616	<0.4	16.2
**Patient 4**	32	1	0	39	None	None	15	NA	1896	NA	160
**Patient 5**	39	1	0	40	None	None	8	130	1683	1.36	80.7
**Patient 6**	37	2	1	35	pPROM and preterm labour	None	9	NA	1740	NA	48.8
**Patient 7**	37	2	1	39	None	None	10	18.7	2575	<0.4	23.5
**Patient 8**	34	3	1	41	None	None	3	NA	2079	NA	6.37
**Patient 9**	30	1	0	40	None	None	20	NA	9266	NA	16.4
**Patient 10**	43	2	1	38	None	None	12	NA	1408	NA	7.31

## References

[1] Kashte S., Gulbake A., El-Amin S.F., Gupta A. (2021). COVID-19 vaccines: Rapid development, implications, challenges and future prospects. Hum. Cell.

[2] Frenck R.W., Klein N.P., Kitchin N., Gurtman A., Absalon J., Lockhart S., Perez J.L., Walter E.B., Senders S., Bailey R. (2021). Safety, Immunogenicity, and Efficacy of the BNT162b2 Covid-19 Vaccine in Adolescents. N. Engl. J. Med..

[3] Shimabukuro T.T., Kim S.Y., Myers T.R., Moro P.L., Oduyebo T., Panagiotakopoulos L., Marquez P.L., Olson C.K., Liu R., Chang K.T. (2021). Preliminary Findings of mRNA Covid-19 Vaccine Safety in Pregnant Persons. N. Engl. J. Med..

[4] CDC Information about COVID-19 Vaccines for Women Who Are Pregnant or Breastfeeding. https://www.portspb.ru/.

[5] Academy of Breastfeeding Medicine ABM Statement. Considerations for COVID-19 Vaccination in Lactation. https://abm.memberclicks.net/abm-statement-considerations-for-COVID-19-vaccination-in-lactation.

[6] ACOG Practice Advice: Vaccinating Pregnant and Lactating PAtients Against COVID-19. December 2020. https://www.acog.org/clinical/clinical-guidance/practice-advisory/articles/2020/12/vaccinating-pregnant-and-lactating-patients-against-covid-19.

[7] Joint Committee on Vaccination and Immunisation: Advice on Priority Groups for COVID-19 Vaccination. 30 December 2020. https://assets.publishing.service.gov.uk/government/uploads/system/uploads/attachment_data/file/950113/jcvi-advice-on-priority-groups-for-covid-19-vaccination-30-dec-2020-revised.pdf.

[8] Perl S.H., Uzan-Yulzari A., Klainer H., Asiskovich L., Youngster M., Rinott E., Youngster I. (2021). SARS-CoV-2–Specific Antibodies in Breast Milk after COVID-19 Vaccination of Breastfeeding Women. JAMA.

[9] DaVanzo R., Moro G., Sandri F., Agosti M., Moretti C., Mosca F. (2020). Breastfeeding and coronavirus disease-2019: Ad interim indications of the Italian Society of Neonatology endorsed by the Union of European Neonatal & Perinatal Societies. Matern. Child. Nutr..

[10] Demers-Mathieu V., Do D.M., Mathijssen G.B., Sela D.A., Seppo A., Järvinen K.M., Medo E. (2021). Difference in levels of SARS-CoV-2 S1 and S2 subunits- and nucleocapsid protein-reactive SIgM/IgM, IgG and SIgA/IgA antibodies in human milk. J. Perinatol..

[11] Sahin U., Muik A., Derhovanessian E., Vogler I., Kranz L.M., Vormehr M., Baum A., Pascal K., Quandt J., Maurus D. (2020). COVID-19 vaccine BNT162b1 elicits human antibody and TH1 T-cell responses. Nature.

[12] Czosnykowska-Łukacka M., Lis-Kuberka J., Królak-Olejnik B., Orczyk-Pawiłowicz M. (2020). Changes in Human Milk Immunoglobulin Profile During Prolonged Lactation. Front. Pediatr..

[13] Egger M., Bundschuh C., Wiesinger K., Gabriel C., Clodi M., Mueller T., Dieplinger B. (2020). Comparison of the Elecsys^®^ Anti-SARS-CoV-2 immunoassay with the EDI™ enzyme linked immunosorbent assays for the detection of SARS-CoV-2 antibodies in human plasma. Clin. Chim. Acta.

[14] Fox A., Marino J., Amanat F., Krammer F., Hahn-Holbrook J., Zolla-Pazner S., Powell R.L. (2020). Robust and Specific Secretory IgA Against SARS-CoV-2 Detected in Human Milk. iScience.

[15] Pace R.M., Williams J.E., Järvinen K.M., Belfort M.B., Pace C.D.W., Lackey K.A., Gogel A.C., Nguyen-Contant P., Kanagaiah P., Fitzgerald T. (2021). Characterization of SARS-CoV-2 RNA, Antibodies, and Neutralizing Capacity in Milk Produced by Women with COVID-19. mBio.

[16] Marchant A., Sadarangani M., Garand M., Dauby N., Verhasselt V., Pereira L., Bjornson G., E Jones C., A Halperin S., Edwards K.M. (2017). Maternal immunisation: Collaborating with mother nature. Lancet Infect. Dis..

[17] Gray K.J., Bordt E.A., Atyeo C., Deriso E., Akinwunmi B., Young N., Baez A.M., Shook L.L., Cvrk D., James K. (2021). Coronavirus disease 2019 vaccine response in pregnant and lactating women: A cohort study. Am. J. Obstet. Gynecol..

[18] Collier A.-R.Y., McMahan K., Yu J., Tostanoski L.H., Aguayo R., Ansel J., Chandrashekar A., Patel S., Bondzie E.A., Sellers D. (2021). Immunogenicity of COVID-19 mRNA Vaccines in Pregnant and Lactating Women. JAMA.

[19] I Mazur N., Horsley N.M., A Englund J., Nederend M., Magaret A., Kumar A., Jacobino S.R., de Haan C., Khatry S.K., LeClerq S.C. (2018). Breast Milk Prefusion F Immunoglobulin G as a Correlate of Protection Against Respiratory Syncytial Virus Acute Respiratory Illness. J. Infect. Dis..

